# Characterization of the Endometrial Microbiome in Patients with Recurrent Implantation Failure

**DOI:** 10.3390/microorganisms11030741

**Published:** 2023-03-14

**Authors:** Francisca Maria Lozano, Belén Lledó, Ruth Morales, Alba Cascales, Mónica Hortal, Andrea Bernabeu, Rafael Bernabeu

**Affiliations:** 1 Instituto Bernabeu Biotech, 03016 Alicante, Spain; 2 Instituto Bernabeu of Fertility and Gynaecology, 03016 Alicante, Spain

**Keywords:** microbiome, microbiota, next generation sequencing, bacterial pathogens, embryo implantation, assisted reproduction, biodiversity, metagenomics

## Abstract

An abnormal endometrial microbiota has been associated with implantation failure; therefore, it may be important to evaluate it in order to improve reproductive outcomes in infertile patients. The main objective of our study was to compare the endometrial microbiome of patients with recurrent implantation failure (RIF) and control patients undergoing assisted reproduction treatment (ART). A prospective cohort study including forty-five patients with their own or donated gametes. The endometrial microbiome was analysed by massive sequencing of the bacterial 16S rRNA gene. Different bacterial communities were detected in RIF and control patients. *Lactobacillus* stands out as the most frequent genus, with 92.27% in RIF patients and 97.96% in control patients, and significant differences were reported between the two groups (*p* = 0.002). No significant differences were found regarding alpha diversity index. In beta diversity analysis, a significant trend was observed in the separation of the bacterial community between established groups (*p* < 0.07). Relative abundance analysis identified genera *Prevotella* (*p* < 0.001), *Streptococcus* (*p* < 0.001), *Bifidobacterium* (*p* = 0.002), *Lactobacillus* (*p* = 0.002) and *Dialister* (*p* = 0.003). Our results demonstrated the existence of an endometrial microbiota characteristic of RIF patients and showed that there might be a relationship between population of the endometrial microbiome and embryo implantation failure, providing us the possibility to improve clinical results in this patients.

## 1. Introduction

Implantation failure is one of the challenges of reproductive medicine, as its causes are often unknown, and effective treatment is rarely available. Approximately 10% of women undergoing in vitro fertilisation (IVF) treatment have this particular problem, creating a distressing and frustrating situation. There is a great need for progress in the development of diagnostic tests to assess the risk of recurrent implantation failure (RIF). Recurrent implantation failure is defined as the failure to achieve a clinical pregnancy after the transfer of at least four good quality embryos in at least three fresh or frozen cycles in a woman under 40 years of age. Detailed studies should be carried out to determine if there is an associated cause [1]. Among the putative factors associated with this condition, there are immunological factors (the presence of antiphospholipid antibodies, certain cytokines or NK cells in maternal serum), genetic alterations in the parents or embryos, uterine abnormalities, or the presence of endometritis. Evaluation of the endometrium, a critical tissue in blastocyst implantation, is essential for a complete assessment of the patient with recurrent implantation failure. Among the causes of implantation failure, various uterine pathologies such as myomas, endometrial polyps, congenital anomalies and intrauterine adhesions must be excluded [2].

These adverse outcomes can result from embryonic or uterine factors since embryo implantation involves a dynamic interaction between the endometrium and the blastocyst. Implantation of the human embryo is a spatiotemporal process involving the intimate association between the embryo and the mother’s endometrium. Implantation requires the correct synchrony and complex interaction at the level of the endometrium, which can be a substantial problem for infertility patients [3]. The causes of embryo implantation failure are challenging because we do not know many of the processes that lead to successful embryo implantation. The most important physiological and molecular mechanisms of implantation are the process of endometrial receptivity, decidualization, trophoblast invasion and blastocyst nidation. Many studies have suggested an association between imbalances in hormone and cytokine levels, alterations in angiogenic and immunomodulatory factors, certain genetic polymorphisms and the occurrence of implantation failure [4]. Uterine analysis should be studied, as microbial colonisation of the upper genital tract has been shown to affect implantation failure, and it is a significant determinant in the success of assisted reproductive treatments (ART).

Technological advances in mass sequencing have made it possible to identify different microbial communities in the uterine cavity and to better understand the ecosystem of endometrial microorganisms. Due to metagenomic analysis of the endometrial microbiome and the application of these new molecular tools, which use the hypervariable regions of the 16S ribosomal subunit, sensitivity has increased significantly [5]. In the uterine microbiota, there are resident bacteria, which maintain homeostasis and health; tourists, which are easily eliminated; or invaders, which facilitate the onset of disease. Studies are needed to further investigate whether there is a “core” or resident uterine microbiota and its contribution to health and homeostasis. In addition, further research is needed to elucidate the functional impact of the uterine microbiota or bacterial species that may be involved as microbial tourists or invaders and the impact these microbes have on endometrial physiology [6]. An imbalance in the balance of microorganisms due to changes in the composition, distribution or functioning of the normal microbiota is known as dysbiosis. Oral probiotics can help restore the vaginal microbiota towards a bacterial balance and achieve a state of eubiosis. Scientific experiments show that the microbiota differs along the reproductive tract, is specific to each woman, and plays an important role in reproductive health [7].

*Lactobacillus* spp. is the most abundant vaginal bacteria in women, which maintain a healthy bacterial community, i.e., a vaginal microbiota dominated by lactobacilli (>90% lactobacilli). These bacteria inhibit the adhesion of other bacteria to epithelial cells and produce lactic acid that kills or inhibits the growth of other bacteria, promoting homeostasis. The ability of lactobacilli to inhibit infection without inducing inflammation may increase pregnancy success in women [8]. Thus, an altered microbiota pattern may predict disease, and this bacterial dysbiosis may lead to negative outcomes for reproductive function [9]. Until now, studies have shown that abnormal vaginal microbiota can negatively affect the clinical pregnancy rate in IVF patients. This negative correlation between abnormal vaginal microbiota and clinical pregnancy rate suggests that it would be desirable to study the uterine microbiota of patients prior to fertility treatment [10]. Currently, an abnormal endometrial microbiota is associated with embryo implantation failure; thus, it is important to assess it to improve reproductive outcomes in infertile patients. A microbiota characterisation study of endometrial fluid and vaginal secretions in infertile women with RIF revealed that the microbiota of endometrial fluid had a higher α-diversity and broader bacterial species than the microbiota of vaginal secretions in both the RIF and control groups. In addition, the endometrial fluid microbiota showed significant variation in community composition between the RIF group and the control group [11]. Thus, research into the effect of an abnormal endometrial microbiome and its correct treatment could improve assisted reproductive techniques.

To advance research of microbial aetiology in the female reproductive tract of patients with implantation failure, the main aim of this research was to identify and assess the endometrial microbiota of RIF and control women undergoing assisted reproductive treatment (ART) using next-generation sequencing (NGS).

## 2. Materials and Methods

### 2.1. Ethics

The Institutional Review Board at Bernabeu Institute authorized our research (reference code: 318/17; date: January 2017), and it was performed in agreement with Helsinki’s declaration. In addition, all patients received information concerning their participation in the study and provided written informed consent.

### 2.2. Study Design and Population

This research included women for in vitro fertility treatment attending our private fertility clinic from May 2017 to May 2019. This prospective cohort study included forty-five patients who underwent assisted reproductive treatment (ART) using their own or donated gametes. Two cohorts of women were included: one cohort with 27 women with recurrent implantation failure (RIF group) and another cohort with 18 women without RIF (control group). The RIF group included women with implantation failure after ≥3 transfer cycles with good-quality embryos. In addition, the women included did not receive any antibiotics during the 3 months prior to fertility treatment. Exclusion criteria were: vaginal infections, uterine malformations, untreated hydrosalpinx, and known recurrent implantation failure factors. Other factors related to RIF were excluded as karyotype or sperm FISH, or positive antiphospholipid antibodies. All study participants were Caucasian females, and they signed the informed consent form.

### 2.3. Sample Collection

During the secretory phase of the cycle prior to frozen embryo transfer (days 18 to 22 of the cycle) endometrial samples were collected. Endometrial sample collection was performed with the Tao Brush IUMC endometrial sampler (Cook Medical, Madrid, Spain). The endometrial sampler was closed in the uterine cavity after endometrial sample collection, preventing contamination by bacteria in the lower vaginal tract. The samples were stored at −80 °C until processing.

### 2.4. Bacterial DNA Extraction

Bacterial genomic DNA extraction from endometrial samples was performed with MagMAX™ CORE Nucleic Acid Purification Kit (Thermo Fisher Scientific, Madrid, Spain) and King-Fisher DUO Prime automated extractor (Thermo Fisher Scientific, Madrid, Spain). DNA was quantified using the Qubit dsDNA High Sensitivity 2.0 kit (Thermo Fisher Scientific, Spain) and the Qubit 4 fluorometer (Thermo Fisher Scientific, Madrid, Spain). Bacterial genomic DNA was stored at −20 °C for further amplification.

### 2.5. V3V4 Hypervariable Region 16S rRNA Gene Amplification

Vaginal and endometrial microbiome patterns were characterized to estimate the classes of bacteria with respect to their prevalence and variability. We studied the bacterial 16S rRNA gene of the samples included in the study by next-generation sequencing (NGS), thus performing metagenomics for the analysis of all samples. During the processing and analysis of the samples, rigorous controls of the reagents used and the equipment were carried out at all stages. Amplification of the V3V4 hypervariable region 16S rRNA gene was performed with Taq DNA polymerase (2x KAPA HiFi HotStart, Roche Diagnostics, Madrid, Spain) in the presence of dNTP, universal primers (357F and 806R) at 1 μM and 100 ng of bacterial DNA, with a final volume of 25 μL. PCR was performed on a thermal cycler (Verity, Applied Biosystems, Foster City, CA, USA). The V3V4 amplicon was visualised on a 1% agarose gel (449 bp) and stored at −20 °C for further sequencing.

### 2.6. Bacterial 16S rRNA Gene Sequencing

The 16S rRNA gene amplicon library was prepared by amplification of the V3-V4 hypervariable region using barcode-specific primers and overhanging adapters. Once obtained, the V3V4 amplicon was purified. Subsequently, the library was generated with the identifying indices of each individual sample using the Nextera XT sequencing kit (Illumina, San Diego, CA, USA), following recommendations from Illumina (16S Metagenomic Sequencing Library Preparation). The indexed libraries were purified and were quantified using a Qubit dsDNA HS 2.0 fluorometer (Thermo Fisher Scientific, Madrid, Spain). Then, libraries were diluted to a stock of 4 nM, pooled and prepared for sequencing. The final stock of the library was 15 pM. MiSeq Reagent Kit v3 (Illumina, San Diego, CA, USA) sequenced the final library. The pooled V3-V4 amplicon library was sequenced using the Illumina MiSeq platform and metagenomics workflow. Patient and control samples were sequenced.

### 2.7. Microbiota Analysis

A bioinformatic analysis of the mass sequencing of V3V4 region 16S rRNA gene of all samples was performed. MiSeq Control Software v2.6 (8 June 2016. Illumina, San Diego, CA, USA) performed primary analysis of sequences obtained from sequencing the 16S gene amplicon library. From the MiSeq system, the unindexed paired-end sequences of independent sample were exported in FASTQ format for further analysis. The QIIME2 package was used for bioinformatics analysis of the study sequences. A total of 114–590 sequences of the 16S rRNA gene were generated from the 45 samples for a mean frequency of 2546 sequences per sample. QIIME2 with Deblur (trim-length 450) performed the analysis of paired endpoint sequences not indexed in FASTA format [12]. Data analysis was performed with MicrobiomeAnalyst MDP [13], Bioconductor Phyloseq [14] and taxonomic characterization with SILVA. Sequences were grouped into taxonomic units (OTU) with a similarity percentage of 97%. To study microbial diversity, an analysis was performed with 1000 sequences per sample for different alpha diversity indexes (Shannon and Simpson indexes). The Shannon index quantifies the types of distinct taxa found in a given community. In addition, this index takes into account species richness and species evenness. If two sites have the same species richness, the more evenly distributed site is considered more diverse than the one dominated by a single species. The Simpson index expresses the probability that two microorganisms are of different species if selected at random in an infinitely large community. Beta diversity expresses the abundance, i.e., the composition of different taxa among the samples studied. Beta diversity analysis is calculated using the UniFrac index. UniFrac uses phylogenetic information, including phylogenetic distances, to measure beta diversity and to compare samples belonging to study groups. In this way, UniFrac relies on the abundance of genera in the samples to measure concordance. PERMANOVA analyses the matrices with beta diversity measures to look for differences in composition according to the group to which they belong. The taxonomic map used a classification based on the filtering of the 99_otus sequence from the SILVA database to the V3V4 region 16S rRNA gene and a fitted classifier classify-sklearn method.

### 2.8. Statistical Analysis

Statistical analysis was performed comparing the endometrial microbiota between RIF and control groups. To test for differences in clinical variables, we used RStudio (v.1.4; R Core Team, 2021). The Chi-square test was used for qualitative variables and the non-parametric test (Mann–Whitney U test) for quantitative variables, considering a *p* < 0.05 as statistically significant. The network was generated by calculating concurrent bacteria genus with significant Pearson correlation coefficients.

## 3. Results

### 3.1. Patient Characteristics

The characteristics of the study subjects, whose mean age was 39.44 years, are shown in Table 1. There was a significant difference in the mean age of the RIF group, 40.44 years vs. 37.94 years in the control group (*p* = 0.003). All women belonging to the RIF group had undergone prior assisted reproduction treatment. However, of the 18 women who did not have implantation failure, 66.67% had undergone prior treatment (*p* = 0.033). There were no significant differences in weight, height, smoking and previous pregnancies in the study population.

### 3.2. Microbial Diversity in RIF and Non-RIF Group

To investigate differences in species diversity between endometrial samples from RIF and non-RIF women, we performed an analysis of the endometrial microbiota.

**Figure 1:**
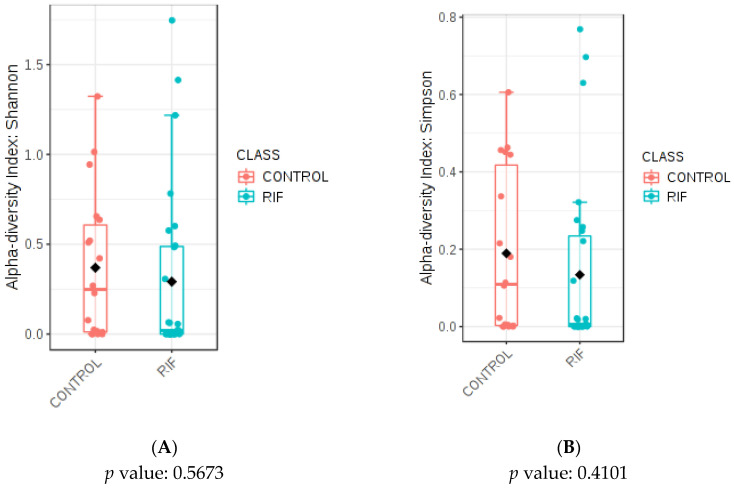
Alpha diversity analysis in the study population. (**A**) Comparative analysis of the Shannon diversity index (*p* = 0.567) and (**B**) the Simpson diversity index (*p* = 0.410) for the RIF and control groups. MicrobiomeAnalyst MDP. Black blocks indicate the midpoint of the alpha-diversity indexes.

Regarding estimating the species richness of the endometrial samples from the study population, alpha diversity was applied at the genus level using two indexes (Figure 1). The analysis of alpha diversity, or the species richness of a particular community that we considered homogeneous, was performed with a rarefaction analysis on 1000 sequences per sample. No statistically significant differences in richness were observed using the Shannon (*p* = 0.56) (Figure 1A) and Simpson (*p* = 0.41) (Figure 1B) indexes.

**Figure 2:**
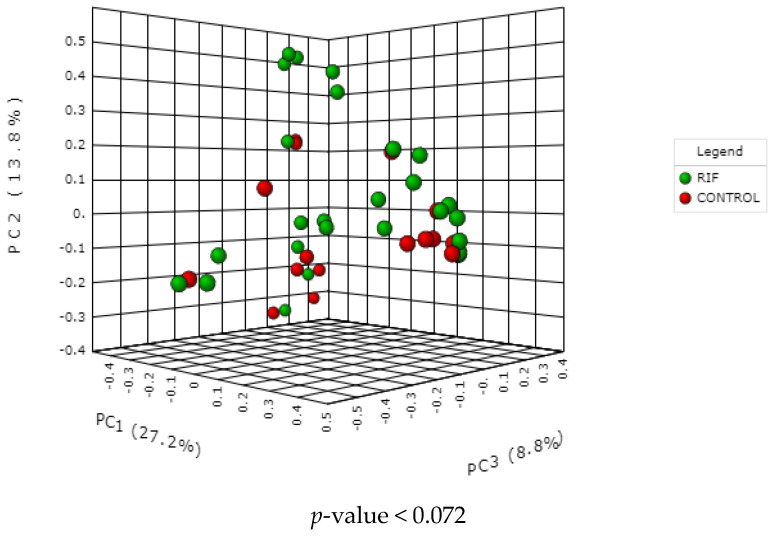
Beta diversity analysis. PCOA showing the clustering between the RIF (green) and control (red) groups, in which each dot represents a sample. PC1 is the principal coordinate component that generates the largest difference in the samples, with a value of 27.02%. PC2 and PC3, with a value of 13.8% and 8.8%, respectively (*p* value < 0.072). MicrobiomeAnalyst MDP.

In order to further show the differences in species diversity between the study samples, a principal coordinate analysis (PCoA) based on the unweighted phylogenetic distances of unifrac at the genus level was performed. Beta diversity or the degree of change or replacement in species composition between different communities was visualised using plots generated by principal coordinate analysis (PCoA). Figure 2 reveals a significant separation in bacterial community composition between the RIF and non-RIF group. When beta diversity of endometrial microbiota was compared by groups, near to significant difference (*p* < 0.07). RIF (green) and control (red) endometrial samples were observed separately in clusters on the three-dimensional PCoA plot.

### 3.3. Relative Abundance Analysis at Genera and Species Level of Endometrial Microbiome Profile RIF vs. Non-RIF Group

**Figure 3:**
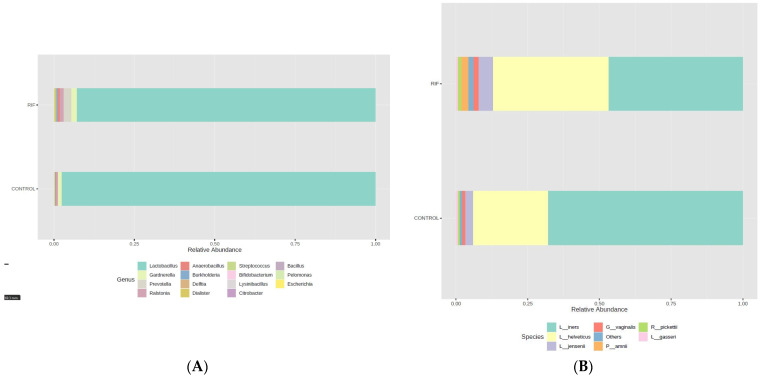
Composition of the bacterial community. Bar chart of the relative frequency of the most abundant genera (**A**) and species (**B**), grouped by RIF and control group. MicrobiomeAnalyst MDP.

The taxonomic profiles of both study groups showed a very different composition of the bacterial community, both at the genus (Figure 3A) and at the species level (Figure 3B). A different relative abundance was observed in the RIF group compared to the control group. Thus, the RIF and control endometrial samples showed a different relative abundance of genera and species, and consequently, of their microbiome profiles. Regarding *Lactobacillus* species composition, in the present study, we observed that the relative abundance of *L. iners* was higher in control samples.

With respect to taxonomic diversity, the most frequent genus was *Lactobacillus* with 92.27% in the RIF group and 97.96% in the control group. We observed a clear difference of the genus *Lactobacillus* abundance between groups (*p* = 0.002). However, the genera compositions were different between the two study groups. In addition, relative abundance analysis identified significant differences in the genera *Prevotella* (0.00% vs. 2.19%; *p* < 0.001), *Streptococcus* (0.05% vs. 0.17%; *p* < 0.001), *Bifidobacterium* (0.11% vs. 0.00%; *p* = 0.002) and *Dialister* (0.062% vs. 0.14%; *p* = 0.003) (Table 2).

Furthermore, at the genus level, *Lactobacillus* was the most abundant and *Prevotella* the least abundant in the control group (Table 2). In addition, *Prevotella* (2.19%), *Gardnerella* (2.18%) and *Ralstonia* (1.15%) were genera enriched in the endometrial microbiota of the RIF group. To understand the different compositions, we investigated the bacterial association in the study groups.

### 3.4. The Bacterial Network of the RIF and Non-RIF Group Genus

**Figure 4:**
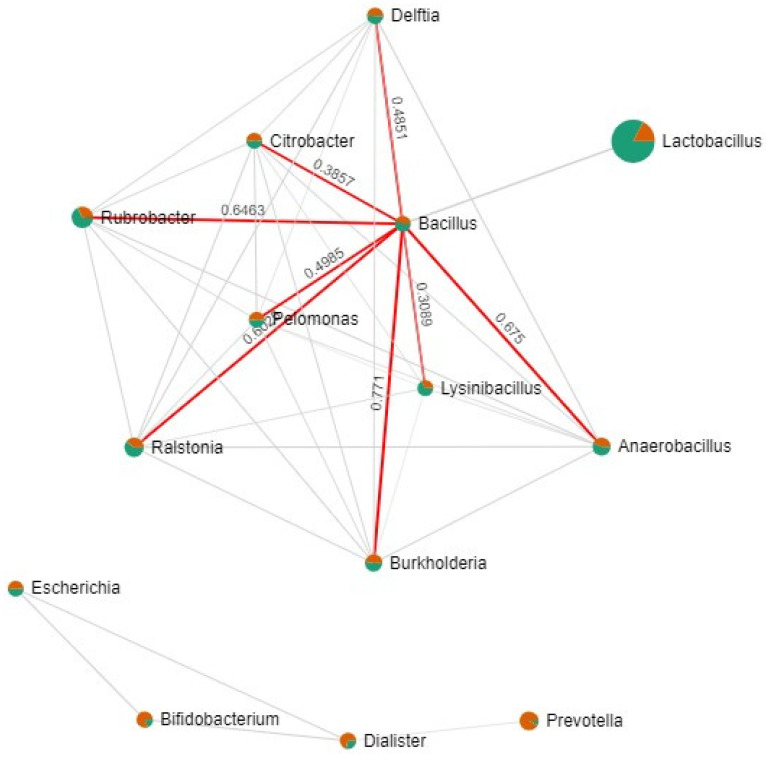
Bacterial network visualising the relationship between genus in all endometrial samples. Circle features: (1) size, proportional to bacterial relative abundances; (2) colour, communities for RIF group (orange) and control group (green). Line features: (1) thickness, from the most significant (thicker) to the less significant correlation (thinner); (2) colour, negative (red) and positive (grey) correlation between the genera. Each concurrent bacterial network was calculated with significant Pearson correlation coefficients.

Figure 4 shows the bacterial association and networks of the endometrial microbiome of RIF and control groups. The concurrent bacterial network visualises the relationship between genera in the endometrial samples from both groups. The relative abundance of bacteria show *Lactobacillus* as the most abundant genus, and it has a negative correlation with all bacteria. *Lactobacillus*, of higher relative abundance in the control group, indicated in green, correlated negatively with a concurrent bacterial network: *Bacillus*, *Delftia*, *Anaerobacillus*, *Citrobacter*, *Gardnerella*, *Ralstonia*, *Burkholderia*, *Pelomonas*, *Lysinibacillus* and much more distantly with a second group of bacteria: *Escherichia*, *Bifidobacterium*, *Dialister* and *Prevotella*.

## 4. Discussion

In our research, we identified the endometrial microbiota and its impact on female reproductive function. According to the results of the present study, the endometrial microbiome of both cohorts was dominated by *Lactobacillus*. An endometrial microbiota with a lower abundance of lactobacilli is associated with recurrent implantation failure (RIF) in infertile women. Thus, there is a characteristic endometrial microbiome in women with embryo implantation failure. Although a trend towards microbial diversity was evident in the RIF cohort compared to the control cohort, no statistically significant differences in richness were observed using any alpha diversity index. As for beta diversity, a difference in bacterial community composition between RIF and control patients was revealed. One of the findings of this study showed that the endometrial microbiome is different between the two cohorts; the relative abundance was reported to be different between the two RIF and non-RIF groups. At the genus level, the abundance of *Lactobacillus* and pathogenic bacteria was different between the two study groups. This difference led us to discover what the bacterial interactions were like in the RIF and non-RIF groups. Thus, our results indicated that *Lactobacillus* was negatively correlated with pathogenic bacteria. Genera associated with implantation failure were more abundant in the RIF group. Thus, our investigations are in agreement with other studies, in which RIF patients with an altered endometrial microbiome could be the reason for their implantation failure [11,15].

Our findings confirm the clinical importance of endometrial dysbiosis. A healthy microbiota is in a state of equilibrium or “eubiosis”; in contrast, disruption of this equilibrium inclines towards a state of “dysbiosis”, where pathogenic bacteria predominate over endogenous bacteria due to inadequate immune response, inflammation or suppressed immune response [16]. In our study, the abundance of *Streptococcus*, *Dialister* and *Prevotella* bacteria has a negative correlation with *Lactobacillus*. Other research also showed a dysbiotic endometrial microbiota profile composed of *Atopobium*, *Bifidobacterium*, *Chryseobacterium*, *Gardnerella*, *Haemophilus*, *Klebsiella*, *Neisseria*, *Staphylococcus* and *Streptococcus* was associated with unsuccessful outcomes. Our results disagree about *Bifidobacterium* relative abundance, and further studies are needed to clarify its effect in RIF patients. Conversely, *Lactobacillus* was consistently enriched in patients with live birth outcomes. A dysbiotic endometrial microbiota profile is known to be associated with poor outcomes; thus, the composition of the endometrial microbiota could be a biomarker for predicting reproductive outcome and improving diagnostic and treatment strategies [17]. Ichiyama et al. [18] also identified specific bacterial communities in the vaginal and endometrial microbiota as biomarkers of implantation failure through a comprehensive analysis of the microbiota in infertile women with a history of recurrent implantation failure.

Distinct microbial communities have been observed in the cervical canal, uterus, fallopian tubes and peritoneal fluid, which differ from those in the vagina. There is a specific continuity in the microbial communities at different points in the female reproductive tract, indicating that it is not a sterile environment. Some studies postulate that the low biomass endometrial microbiota is determined by specific colonisation of the endometrium, mainly by bacteria ascending from the vagina [19]. Pathogenic bacteria found in the endometrium of women with implantation failure are taxonomically similar to those found in the lower genital tract of women with bacterial vaginosis, which is a risk factor for miscarriage, spontaneous preterm delivery, intra-amniotic infection, puerperal endometritis and adverse perinatal outcomes [20]. Thus, impaired microbiota communities containing specific bacteria in both the endometrium and vagina are associated with implantation failure [21]. Our results also suggest that implantation failure may also be caused by ascending pathogens and that the composition of an endometrium with a lower abundance of lactobacilli may be the cause of an increased risk of negative implantation outcomes in RIF patients. In the group of patients with RIF, the abundance of vaginosis-related bacteria and the scarcity of protective bacteria, which produce lactic acid and lower pH, could be the cause of an increased risk of adverse implantation outcomes. Wee et al. [22] conducted a retrospective pilot study to establish if the microbiota of the female reproductive tract differed among infertile and fertile women. Infertile women tended to have a higher frequency of *Ureaplasma* in the vagina and *Gardnerella* in the cervix. This finding has been confirmed by a recent study, which suggests that a considerable percentage of non-*Lactobacillus*-dominated microbiota (NLD) was found in the endometrium of infertile Japanese women. Thus, increasing the endometrial level of lactobacilli to >90% could favour the implantation outcome of infertile NLD patients [23]. In summary, we know that altered uterine microbiota in which lactobacilli do not predominate is associated with reproductive failure and adverse pregnancy outcomes: early pregnancy loss, late miscarriage and preterm delivery [24]. Infertility treatment is inherently complicated; however, the importance of the implantation phase has been shown to be critical to positive outcomes. A *Lactobacillus*-dominated endometrium is more receptive than an endometrium with a high bacterial diversity and a low proportion of *Lactobacillus* ratio. Understanding how to assess and diagnose microbiome dysbiosis in the female reproductive tract could improve reproductive outcomes [25].

Several investigations have recently defined the resident endometrial microbiota. However, there is no consensus on the bacterial core of the endometrium and its impact on the reproductive tract in terms of fertility and pregnancy outcomes. The lack of consensus may be due to the absence of large cohorts in microbiome studies. Therefore, there is a need for comparability between studies and the establishment of general protocols that allow for this type of analysis. Future studies that understand the mechanisms of microbiota–host interactions could shed light on how bacterial communities influence infertility. A healthy physiological endometrial microbiota is a set of microorganisms permissive for embryo implantation, regardless of the minimal presence of pathogenic bacteria [26]. Understanding the healthy endometrial microbiota could help to develop diagnostic as well as personalised therapies for the prevention of obstetric complications and personalised treatments through nutritional, microbiotic or pharmaceutical interventions [27]. Kyono et al., in 2019 [28], demonstrated that probiotics and oral antibiotics are a good therapy to improve pregnancy rates prior to IVF embryo transfer. Women with non-*Lactobacillus*-dominated microbiota were treated with antibiotics and probiotics and were successfully converted to *Lactobacillus*-dominated microbiota. Oral probiotics are live microorganisms, which when administered in adequate amounts, confer a health benefit to the host, restore the state of eubiosis and consequently improve female fertility. Restoring the microbiota to a healthier one is an opportunity to improve in vitro fertilisation (IVF) success rates and outcomes in these patients. Lactic acid bacteria prevent host diseases such as bacterial vaginosis, yeast vaginitis, urinary tract infection and sexually transmitted diseases. Probiotic administration may be important to maintain normal urogenital health as well as to prevent or treat infections [29].

In terms of *Lactobacillus* species composition, the relative abundance of *Lactobacillus iners* is higher in samples from patients without implantation failure, although it is also the most abundant *Lactobacillus* species in samples from patients with implantation failure. *L. iners* is a prevalent bacterial species of the vaginal microbiome, which possesses many probiotic characteristics in its contribution to the maintenance of a healthy vaginal microbiome. It is known to be present in a healthy vagina and is recovered in large numbers in bacterial vaginosis (BV). One possible explanation for the difference in *L. iners* species between the samples studied is that it is a transient species that colonises after the vaginal environment has been altered, but also appears to be an opportunistic pathogen. Consequently, *L. iners* is a unique species of *Lactobacillus* that is often classified as a transient species that colonises the vagina following ecological disturbance. However, it remains controversial whether *L. iners* is beneficial or pathogenic to the host microbiome [30].

We studied the microbial aetiology in the female reproductive tract of patients with RIF, with the aim of finding an appropriate diagnosis and treatment for implantation failure. Evidence supports that implantation failure may be due to several different factors such as the maternal immune system, embryo and parental genetics, anatomical factors, haematological factors, the microbiome of the reproductive tract, and the endocrine environment, which influence embryo and endometrial synchrony [31]. It is possible that resident populations of microorganisms at the endometrial level interact with the endometrial epithelium and/or alter endometrial expression of leukocytes and cytokines. Therefore, these events, either in isolation or acting together, may impair endometrial receptivity and affect adequate implantation [27]. The microbiota may be one piece of a complex mechanism that contributes to the interplay of hormones, immune cells and physiological adaptations necessary for a successful pregnancy. The microbiome, immunity, endocrinology in pregnancy and foetal development need to be studied together to obtain a more complete picture. It is therefore necessary to study reproductive immunology and the microbiota involved together [32].

New technologies have enhanced the metagenomic analysis of samples that allow us to further characterise the microbiome of the reproductive tract [33]. We have better characterised the normal and abnormal endometrial microbiome and determined that sites previously thought to be sterile actually have robust microbiomes. Knowledge of the microbiome in human health and disease has been reported, especially in relation to human reproduction. As the dysbioses of the reproductive tract become better characterised and understood, we will be better equipped to manipulate it with greater precision [34]. Whether the endometrial microbiome is a new hope in reproductive medicine is currently in question, and the applicability of microbial composition and molecular biomarkers needs to be confirmed. Therefore, identification of the microbial profile in health and disease should be addressed before offering any treatment strategy to patients for successful embryo implantation and establishment of pregnancy. Future studies will clarify to what level endometrial microbes contribute to embryo implantation and pregnancy establishment, and how the microenvironment might be modified in endometrial microbial dysbiosis [35].

The strength of our study is that it contributes to the identification of endometrial dysbiosis, affecting the embryo implantation process by comparing the endometrial microbiota of women with and without implantation failure. It confirms the importance of assessing the endometrial microbiota in infertile patients, its correct treatment with antibiotics and/or probiotics for the normalisation of the microbiota and its consequences in terms of clinical outcome of IVF. The main limitation is the small size of the study population; thus, future larger studies are needed to confirm our results on microbiota in embryo implantation failure. Assessing the endometrial microbiome is complex because it involves the detection of a low-biomass microbiota. Cohort characteristics such as ethnicity and age are other main limitations of studies of the endometrial microbiome, as they may be responsible for community differences in the female genital tract.

## 5. Conclusions

This study demonstrated that women with embryo implantation failure have a characteristic endometrial microbiome. The profile of the endometrial microbiome of implantation failure showed a lower relative abundance of *Lactobacillus*, and other genera such as *Prevotella*, *Streptococcus* and *Dialister* were identified. In beta diversity, a trend towards separation of the bacterial community between cohorts was observed. The endometrial microbiota showed different relative abundance in women with a history of implantation failure. The concurrent bacterial network demonstrates the bacterial association between genera in the endometrial samples of the RIF and non-RIF groups. *Lactobacillus* as the most abundant genus is negatively correlated with pathogenic bacteria. In addition, several genera associated with implantation failure were more abundant in the RIF group: *Streptococcus*, *Dialister* and *Prevotella*. Our results contribute to understanding why these women with different patterns of endometrial microbiome have recurrent implantation failure, suggesting that disturbance in microbiota communities containing specific bacteria in the endometrium may be a biomarker of implantation failure. Our interest is to understand and analyse the reproductive microbiome in relation to fertility, with the aim of helping patients with embryo implantation failure who have undergone assisted reproductive treatment.

## Figures and Tables

**Table 1:** Characteristics and clinical outcomes of study population. *p* values < 0.005 are shown in bold.

	Total	Control	RIF	*p* Value
N	45	18	27	
**Age (years) ± SD**	39.44 ± 3.89	37.94 ± 3.51	40.44 ± 3.88	**0.0305**
**Weight (kg) ± SD**	67.5 ± 23.47	57.86 ± 8.82	71.25 ± 26.40	0.0705
**Height (m) ± SD**	157.67 ± 21.30	157.29 ± 8.42	157.80 ± 24.46	0.9570
**Tobacco user (%)**	13.3	16.67	11.21	0.5912
**Previous pregnancies (%) ± SD**	0.69 ± 0.60	0.72 ± 0.75	0.67 ± 0.48	0.7832
**Previous treatments (%)**	77.78	66.67	100	**0.0332**

**Table 2:** Taxonomic diversity of the bacterial community at the genera level in the control and RIF cohorts.

Genus	Control	RIF	*p* Value
*Lactobacillus*	97.9634%	92.2742%	0.002
*Prevotella*	0.0000%	2.1911%	<0.001
*Gardnerella*	1.0697%	2.1804%	
*Ralstonia*	0.4459%	1.1598%	
*Anaerobacillus*	0.2195%	0.6281%	
*Bifidobacterium*	0.1122%	0.0000%	0.002
*Burkholderia*	0.1097%	0.4461%	
*Dialister*	0.0620%	0.1491%	0.003
*Delftia*	0.0554%	0.2284%	
*Streptococcus*	0.0520%	0.1780%	<0.001
*Lysinibacillus*	0.0358%	0.0357%	
*Bacillus*	0.0162%	0.0321%	
*Citrobacter*	0.0139%	0.0678%	

## Data Availability

Data are not available due to ethical or privacy restrictions.
